# Deciphering the role of DNA methylation in multiple sclerosis: emerging issues

**DOI:** 10.1007/s13317-016-0084-z

**Published:** 2016-09-07

**Authors:** Maria Sokratous, Efthimios Dardiotis, Zisis Tsouris, Eleni Bellou, Amalia Michalopoulou, Vasileios Siokas, Stylianos Arseniou, Tzeni Stamati, Georgios Tsivgoulis, Dimitrios Bogdanos, Georgios M. Hadjigeorgiou

**Affiliations:** 1 Department of Neurology, Laboratory of Neurogenetics, University of Thessaly, University Hospital of Larissa, Larissa, Greece; 2 Second Department of Neurology, University of Athens, School of Medicine, “Attikon” University Hospital, Athens, Greece; 3 Department of Rheumatology and Clinical Immunology, Faculty of Medicine, School of Health Sciences, University General Hospital of Larissa, University of Thessaly Viopolis, 40500 Larissa, Greece; 4 Cellular Immunotherapy and Molecular Immunodiagnostics, Biomedical Section, Center for Research and Technology-Hellas (CERTH)-Institute for Research and Technology-Thessaly (IRETETH), 41222 Larissa, Greece

**Keywords:** DNA methylation, Epigenetics, Multiple sclerosis, Methylocytosine, Autoimmunity

## Abstract

Multiple sclerosis (MS) is an autoimmune inflammatory and neurodegenerative disease of the central nervous system that involves several not yet fully elucidated pathophysiologic mechanisms. There is increasing evidence that epigenetic modifications at level of DNA bases, histones, and micro-RNAs may confer risk for MS. DNA methylation seems to have a prominent role in the epigenetics of MS, as aberrant methylation in the promoter regions across genome may underlie several processes involved in the initiation and development of MS. In the present review, we discuss current understanding regarding the role of DNA methylation in MS, possible therapeutic implications and future emerging issues.

## Epigenetics in MS: convergence between genetic and environmental factors

Multiple sclerosis (MS) is a complex inflammatory and degenerative disease of the central nervous system (CNS) that involves several not yet fully elucidated pathophysiologic mechanisms and evidences of implication of both environmental and genetic factors [1]. Epigenetics may be the bridge between genotypes, environmental exposures, and phenotypes.

Epigenetic modifications are heritable, reversible alterations in gene expression, which do not affect gene sequence. They depend on environmental and biological conditions, resulting in different cell responses [2]. The main epigenetic mechanisms consist of DNA methylation, histone alteration, and micro-RNAs. DNA methylation aims to prevent transcription factors from binding to gene promoter, thus silencing gene expression. This procedure is achieved by DNA methyl transferases (DNMTs), which convert cytosine of CpG islands in gene promoters into 5-methylcytosine. Histone alterations include several procedures that regulate transcription, such as acetylation and phosphorylation. Acetylation of the N-terminal tail of a histone leads to decompression of chromatin and upregulates transcription [3]. Micro-RNAs are single stranded, small in nucleotide amount RNAs, which do not encode any proteins. They can affect gene expression, acting after gene transcription and their expression depends on the interaction with the other two mechanisms [4]. Micro-RNAs silence gene expression by improper binding to a specific or to multiple mRNA sequences into a ribonucleoprotein called RISC (RNA-associated silencing complex). Even the same mRNA sequence can be regulated by several micro-RNAs bound up to it [5]. Epigenetic mechanisms seem to influence the development of numerous diseases, such as systemic lupus erythematosus (SLE), cancer, rheumatoid arthritis (RA), and diabetes melitus type 1 (T1DM) [6–8].

In MS, a great effort of collaborative studies has been made in the last decade to define the genetic architecture of MS. These efforts have yielded, till now, 110 genetic risk factors of MS [9]. However, these variants along with HLA loci can account only for about 27 % of the apparent MS heritability [10], highlighting the possible role of interactions between environmental and genetic factors [11]. In addition, genetic factors are unable to explain the low MS concordance rate between monozygotic twins only by themselves [12]. Moreover, “gender bias,” with a higher prevalence of MS in females could be attributed to defective regulation of X chromosome through epigenetic effects, or to the parent-of-origin effect, where descendants of diseased mothers have greater chance of developing MS than offspring of diseased fathers, caused probably by epigenetic mechanisms such as genome imprinting [13].

In addition, epigenetics may be the link between environmental factors and susceptibility to MS. The specific MS geographical distribution and the results from migration studies are believed to epigenetically modify MS susceptibility [14]. Low serum vitamin D, which influences MS course and disability, seems to affect the expression of histone-modifying enzymes [15]. Smoking, which may be associated with MS in a dose dependent pattern [16], has been linked to DNA methylation and other epigenetic modifications [17]. Finally, epigenetic control of human endogenous retroviral family type W (HERV-W) and Epstein–Barr virus (EBV) elements that are present in the human genome may be critical in the development and evolution of MS [18, 19].

## DNA methylation

DNA methylation is assumed to be among the best described epigenetic mechanisms, first referred to its correlation with cancer, regulating the expression of oncogenes and tumor suppressor genes [20]. However, over the past decade, a huge effort has been made to explain its role in immunity and autoimmunity. To begin with, DNA methylation is an essential process for normal cell development, proliferation, and genome integrity [21]. It is mediated by a number of enzymes called DNA methyltransferases (DNMTs), the most important of which are DNMT1, DNMT3a, and DNMT3b. These enzymes are responsible for the positioning of a methyl group at the 5′-carbon position of a cytosine converting it into 5′-methylcytosine. The first one is associated with the preservation of methylation to the daughter strands during every replication cycle, while the other two enzymes set up methylation de novo at the early developmental stages of cell life. DNA methylation mainly occurs at regions where a guanine accompanies the cytosine, forming a dinucleotide. Hundreds of these dinucleotides are found repetitively in gene promoters, as CpG islands. Hypermethylation of these sites leads to silencing of the gene, by not allowing transcription factors to bind to the gene promoter, while hypomethylation to the transcription and usually to the expression of the subjected gene [22]. The demethylation of those regions can easily occur in either a passive or in an active way. The passive one is favored during DNA replication, while the active one is achieved by other enzymes not particularly during cell division. Methylation patterns usually pass to the next generation through meiosis and also form the chromatin structure, affecting cell function [23].

## The role of DNA methylation in MS

The majority of attempts to identify changes in DNA methylation across the genome can be distinguished into two approaches: candidate-gene approach, where specific genetic loci are selected and examined for differences in DNA methylation and through genome-wide methylation analysis. Also combinations of these techniques can also be applied [24]. The effect of DNA methylation has been studied in mouse models of MS, especially in mice with experimental autoimmune encephalomyelitis (EAE), which have been proved very promising in extrapolating these results to human MS patients [25]. Quite a few studies have also analyzed the effect of aberrant DNA methylation on MS phenotypes, using various approaches in sample size, definitions, methodology, and statistical approach, and they have come upon various results. These studies are presented in Table 1.

**Table 1:** Baseline characteristics of studies about DNA methylation and MS

Author [references]	Tissue	Sample	Type of study	Results
Mastronardi [28]	Cortical white matter of brain and thymus	Normal controls (*n* = 4) MS patients (*n* = 12)	Candidate-gene DNA methylation approach	30 % demethylation in PAD2 gene promoter only in the white matter of MS patients
Ramagopalan [31]	RBMCs	50 pairs of monozygotic discordant twins with MS (68 % RRMS)	Candidate-gene DNA methylation approach	No significant association between MS and methylation level of MCH2TA promoter IV
Baranzini [33]	CD4+ T lymphocytes	3 pairs of discordant MS twins (2 RRMS and 1 SPP)	Genome-wide DNA methylation approach	No evidence of epigenome differences
Handel [32]	PBMCs	Benign MS (*n* = 48) malignant MS (*n* = 20)	Candidate-gene DNA methylation approach	No significant association between DNA methylation across HLA-DRB1*1501, HLA-DRB5 and MS severity
Ligget [34]	cfpDNA	RRMS(r) (*n* = 30) RRMS(e) (*n* = 29) healthy controls (*n* = 30)	Genome-wide DNA methylation approach	Differences in DNA methylation: in 15 promoters between RRMS(r) patients and healthy controls, in 14 promoters between RRMS(c) patients and healthy controls and in 5 promoters between RRMS(r) patients and RRMS(c) patients. CDKN2B gene displayed the most differentially methylated pattern [71.0 % methylation in RRMS(r) and 22.6 % in controls]
Janson [35]	CD4+ T lymphocytes	7 healthy controls (*n* = 7) 10 RRMS patients (*n* = 17) [under natalizumab (*n* = 10), without treatment (*n* = 2), under glatiramer acetate (*n* = 3), under IFN-1b (*n* = 2)]	Candidate-gene DNA methylation approach	DNA hypomethylation in FOXP3 and IL-17 genes, in MS patients under no natalizumab therapy compared to healthy controls
Calabrese [29]	PBMCs	Healthy controls (*n* = 30) MS patients (*n* = 32) [RRMS (*n* = 31), SPMS (*n* = 1)]	Candidate-gene DNA methylation approach	Upregulation and overexpression of PAD2 gene promoter due to hypomethylation. No correlation with MS disease duration, EDSS, MRI activity in the entire sample or after stratification by gender. Mild correlation between PAD2 concentration in peripheral blood and EDSS, revealed by 63 % of the patients. No significant results for PAD4
Kumagai [36]	Peripheral blood leukocytes (293T cells)	Normal subjects (*n* = 19) MS subjects (*n* = 69) [PPMS (*n* = 7), RRMS (*n* = 50), SPMS (*n* = 12)]	Candidate-gene DNA methylation approach	Increased level of methylation in promoter 2 of SHP-1 in MS patients compared to healthy controls. No association between methylation in SHP-1 promoter and MS type, years of disease and EDSS score
Calabrese [39]	PBMCs	Healthy controls (*n* = 40) MS patients (*n* = 40)	Candidate-gene DNA methylation approach	Downregulation of TET2 and DNMT1 gene expression in MS PBMCs induced by defective methylation
Graves [38]	CD4+ T lymphocytes	Healthy controls (*n* = 28) RRMS patients (*n* = 30)	Genome-wide DNA methylation approach	Differences in DNA methylation in 38 different genes (19 within MHC region, 55 at non-HLA genetic loci). Highest signal at 6p21.32 of HLA-DRB1. Strong association between DNA methylation of HLA-DRB1 and HLA-DRB1 haplotype
Huynh [43]	Brain tissue	Healthy controls (*n* = 19) MS patients (*n* = 28)	Genome-wide DNA methylation approach	220 hypomethylated DMRs (containing 1235 CpGs) and 319 hypermethylated DMRs (containing 1292 CpGs) revealed, with oligodendrocyte-specific genes and genes regulating oligodendrocyte survival among them
Bos [44]	Whole blood, CD4+ and CD8+ T lymphocytes	Healthy controls (*n* = 14) RRMS patients (*n* = 14) all females	Genome-wide DNA methylation approach	Differences among CD4+, CD8+ and WB cells in their overall DNA methylation. No consistent DNA methylation differences between MS and controls. Difference in methylation level of Forty CpG-sites between MS patients and controls. The most significantly associated sites: a probe near TMEM48 transcription start site, another probe in the first exon of APC2 and several CpG-sites within DNHD1 gene. Increased hypermethylation in CD8+ cells in patients with disease duration over 7 years or lower, compared to those with duration above 8 years
Maltby [45]	CD8+ T lymphocytes	Healthy controls (*n* = 28) RRMS patients (*n* = 30)	Genome-wide DNA methylation approach	79 methylated CpGs detected in genes outside MHC locus, not previously related to MS. No overlap of sites with methylation changes between CD4+ and CD8+ T cells (compared with previous results). Strong association between methylation changes in FTL, ERG and DCAF4 genes and MS
Neven [46]	Whole blood	Healthy controls (*n* = 137) RRMS patients (*n* = 51)	Genome-wide DNA methylation approach (repetitive elements)	Hypermethylated Alu, LINE-1 and SAT-α repetitive elements in MS patients compared to healthy controls. Higher disability associated with hypomethylation in LINE-1 and hypomethylation in Alu. No significant association between methylation and MS course, age of MS onset, multisystem disorders, presence or amount of CSF oligoclonal bands and spinal cord relapse

*MS* multiple sclerosis, *PPMS* primary progressive multiple sclerosis, *SPMS* secondary progressive multiple sclerosis, *RRMS* relapsing-remitting multiple sclerosis, *RRMS* (*r*) relapsing-remitting multiple sclerosis in remission, *RRMS* (*e*) relapsing-remitting multiple sclerosis in exacerbation, *MRI* magnetic resonance imaging, *EDSS* expanded disability status scale, *RBMCs* peripheral blood mononuclear cells, *PAD2* peptidylarginine deiminase 2, *PAD4* peptidylarginine deiminase 4, *cfpDNA* cell-free plasma DNA, *CSF* cerebrospinal fluid, *IFN* interferon, *MCH* major histocompatibility complex, *HLA* human leukocyte antigen, *CDKN2B* cyclin dependent kinase inhibitor 2B, *IL* interleukin, *FOXP3* forkhead box P3, *SHP*-1 protein-tyrosine phosphatase SHP-1, *DNMT1* DNA methyltransferase1, *TET2* ten-eleven translocation methylcytosine dioxygenase 2, *DMR* differentially methylated region, *FTL* ferritin light chain, *ERG* ETS-related gene, *DCAF4*, *DDB1*- and *CUL4*-associated factor 4, *THEM48* transmembrane protein 48, *APC2* adenomatous polyposis coli protein 2, *DNHD1* dynein heavy chain domain 1, *MHC2TA* class II transactivator gene promoter IV

*Peptidylargininedeiminase 2 (PAD2) and 4 (PAD4)* are enzymes expressed in the brain as well as in peripheral blood cells. Upregulation of PAD2 and PAD4 genes may contribute to deamination of myelin basic protein (MBP) and to consequent loss of immune tolerance in MS patients [26, 27]. PAD2 gene hypomethylation at a rate of 30 % of the cytosines has been reported in the white matter of MS patients, due to increased activity of DNA demethylase [28]. Moreover, authors suggested that the observed demethylation is tissue specific (in the white matter) and a characteristic feature only of MS. They observed no significant hypomethylation in the thymus gland of MS patients or in the white matter of patients with other neurological diseases (Alzheimer’s, Huntington’s, and Parkinson’s diseases) [28]. However, PAD2 gene has also been found to be upregulated and overexpressed in peripheral blood mononuclear cells (PBMCs), an upregulation that is also associated with hypomethylation of CpGs of PAD2 promoter [29]. PAD2 overexpression has not been correlated with MS disease duration, gender, expanded disability status scale (EDSS), and magnetic resonance imaging (MRI) activity. However, in a cluster of 63 % of the MS subjects, a mild correlation between PAD2 concentration and EDSS in peripheral blood has been revealed [29]. Research about PAD4 gene has not showed any significant alteration in the methylation status of PAD4 in peripheral blood tissue of MS patients [29]. Changes in DNA methylation of PAD2 promoter may lead to upregulation of PAD2 gene and increased production of PAD2 protein, which, in turn, regulates the production of citrullinated MBP. This less stable form of MBP leads to myelin destabilization and activation of immune response during MS course [28].

There is considerable amount of evidence to support the association between the major histocompatibility complex (*MHC)* class II and MS [11]. Furthermore, the expression of MHC molecules is regulated by MHC2TA transactivator, which, in turn, is influenced by methylation of its gene promoter IV [30]. A study aiming to elucidate the possible contribution of the methylation level of MCH2TA promoter IV on MS susceptibility was conducted, without revealing any significant association [31]. In an attempt to identify the contribution of epigenetic changes (inactivation) of *HLA*-*DRB5 and/or HLA*-*DRB1* to the severity of MS, MS patients were classified according to MS severity, based on EDSS score and MS type [32]. An additional stratification was also made according to homozygosity and heterozygosity for HLA-DRB 1*1501 [32]. However, the study showed no difference in DNA methylation at CpG dinucleotides across HLA-DRB1*1501 and HLA-DRB5, neither between the entire malignant and entire benign groups, nor between HLA-DRB1*1501 positive malignant and HLA-DRB1*1501 positive benign subjects [32]. However, a marginal higher proportion of methylated DNA among HLA-DRB1*1501 heterozygous MS patients with malignant phenotype compared to the benign one was detected. On the contrary, a lower amount of DNA methylation in HLA-DRB1*1501 homozygotes with malignant MS was found, compared with HLA-DRB1*1501 homozygotes with benign MS [32].

In a very interesting study, Baranzini and his colleagues examined three pairs of discordant MS twins for possible changes in methylation level in CD4 + T lymphocytes using a genome-wide DNA methylation approach. Surprisingly, no significant epigenome differences were detected [33].

Another study aimed to elucidate the role of methylation in *cell*-*free plasma DNA (cfpDNA)*, which is found in human plasma in the form of heterogeneous polynucleotides in MS [34]. Differences in DNA methylation appeared in several gene promoters and more precisely: in 15 promoters when relapsing-remitting multiple sclerosis in remission [RRMS(r)] was compared to healthy controls, in 14 when relapsing-remitting multiple sclerosis in exacerbation [RRMS(e)] was compared to healthy controls, and in 5 when RRMS(r) was compared with RRMS(e). The most differentially methylated gene was found to be the cyclin dependent kinase inhibitor 2B (*CDKN2B*) (71.0 % methylation in RRMS(r) and 22.6 % in controls) [34].

DNA demethylation has also been examined in *IFNG, FOXP3, IL*-*13 and IL*-*17* genes that represent key regulators of immune response and Th cell differentiation [35]. MS patients under no natalizumab treatment compared to healthy controls were found to have DNA hypomethylation of FOXP3 and IL-17 genes in isolated CD4+ T cells. However, this finding was not present in MS patients under natalizumab treatment. The authors suggested that hypermathylated DNA in MS patients treated with natalizumab may not be a consequence of the drug [35].

Protein-tyrosine phosphatase *SHP*-*1* may be implicated into immune system activation and inflammatory demyelination of MS patients [36]. MS patients were found to have a higher methylation level at the promoter 2 of SHP-1 gene and, consequently, decreased SHP-1 expression and increased leukocyte-mediated inflammation [36]. SHP-1 promoter gene methylation has previously been reported to repress the transcription of the SHP-1 gene in lymphoblastoid cells [37]. However, no association between methylation of the SHP-1 promoter and MS type, years of disease or EDSS score was detected [36]. Hypermethylation in a CpG island of SHP-1 has also been identified by a genome-wide DNA methylation study [38].

Regulation and expression of *DNMT1, DNMT3a, DNMT3b, TET1, TET2, and TET3* genes influence the function of the corresponding enzymes, which are implicated in the conversion of 5-methylcytosine (5mC) into 5-hydroxymethylcytosine (5hmC) [39, 40]. Calabrese et al. investigated the methylation status of these genes in MS, by isolating PBMCs from MS patients and normal controls. They detected alterations in the methylation level of DNMT1 and TET2 gene promoters [39]. These methylation changes were accompanied by a downregulation of DNMT1 and TET2 levels in MS subjects. In addition, a moderate negative correlation was found between TET2 expression and MS duration [39]. A significant downregulation of TET3 genes in secondary progressive multiple sclerosis (SPMS) patients has also been observed [41].

A genome-wide DNA methylation analysis revealed 19 CpG islands within MHC region. The 6p21.32 of HLA-DRB1 locus was found to be the most significantly related to RRMS [38]. Moreover, DNA methylation of HLA-DRB1 seems to depend on the pattern of HLA-DRB1*1501 haplotype [38]. The study also revealed significant differences in methylation status of 55 CpGs islands of non-HLA genetic loci, the majority of which (30 out of 55) had been previously linked to MS [38]. However, most of non-HLA genes that were linked to MS [42] did not reveal any change in DNA methylation [38].

DNA methylation status in *genes linked to oligodendrocyte function and immune response* in brain areas free of inflammation and demyelination was linked to MS. In a genome-wide DNA methylation approach, MS duration was associated with the differentially methylated regions (DMRs). The analysis identified 220 hypomethylated DMRs (containing 1235 CpGs) and 319 hypermethylated DMRs (containing 1292 CpGs), after correction for predictors that influence the methylation status. Remarkably, DNA hypomethylation at CpGs was distributed at a surrounding of transcriptional start sites, while hypermethylated CpGs were mainly located in the main body of genes. Among the hypermethylated autosomal genes, several oligodendrocyte-specific genes, such as myelin basic protein (*MBP*) and sex determining region Y-box 8 (*SOX8*), as well as genetic loci that regulate survival, such as N-myc downstream regulated 1 (*NDRG1*) and bcl-2-like protein 2 (*BCL2L2*), were reported to be hypermethylated. To avoid any disarray, due to inactivation of X chromosome, researchers also performed a gender-specific analysis of the X chromosome. Analysis of a female subgroup revealed different cluster, including hypermethylation in neighboring gap junction protein beta 1 (*GJB1*), a gene that may be involved in the function of oligodendrocyte. Upregulated transcripts in MS compared to controls were detected in cathepsin Z (*CTSZ*) vandlegumain (*LGMN*), which are implicated to immune system regulation and biological functions of the nervous cells. Downregulation was observed in cryptochrome circadian clock 2 (*CRY2*), which influences the circadian rhythm and in *BCL2L2,* which may regulate neuronal death and the survival of oligodendrocytes. The difference in DNA methylation, according to methylation status (hyper- or hypo-) and the average methylation difference between MS and controls, was validated by an independent sample. It was shown that *CTSZ* and hydroxyacylglutathione hydrolase-like (*HAGHL*) genes were differently expressed in MS compared to controls. In a third independent subgroup, *BCL2L2* differences in expression did not reach statistical significance [43].

Another genome-wide DNA methylation approach detected differences in the overall DNA methylation among CD4+, CD8+, and whole blood (WB) cells, although no consistent DNA methylation differences between MS and controls were observed [44]. Forty CpG-sites exhibited differences in their methylation level between MS patients and controls, while the strongest associations were in a probe near transmembrane protein 48 (*TMEM48*) transcription start site, in the first exon of adenomatous polyposis coli protein 2 (*APC2*) and in several CpG-sites within dynein heavy chain domain 1 (*DNHD1*) gene [44]. In MS patients, CD8+ T cell DNA revealed strong evidence for hypermethylation in a few CpG-sites. Moreover, higher hypermethylation in CD8+ has been found in patients with disease duration over 7 years or lower, compared to those with duration above 8 years [44].

A recent genome-wide association study revealed 79 methylated CpGs in genes outside MHC locus (not previously related to MS). Both CD4+ and CD8+ T cells had a single hypermethylated CpG in the MORN repeat-containing protein 1 (*MORN1*) gene but at different genetic sites of the gene. Ferritin light chain (*FTL*) gene was the most significantly associated promoter revealing DNA hypomethylation in MS patients compared to controls. In addition, ETS-related gene (*ERG*), DDB1-, and CUL4-associated factor 4 (*DCAF4*) were found to be hypermethylated in MS patients compared to healthy controls [45]. Changes in FTL gene expression could influence the load of iron deposits in the gray matter of patients with RRMS, while misregulation of the transcript of ERG could influence apoptosis, cell proliferation and inflammation procedures. What is more, the defective function of DCAF4 could be implicated in neurodegeneration [45].

Differential methylation status of *Alu, LINE*-*1 and SAT*-*α repetitive elements*, widely known as estimators of global DNA methylation, may also contribute to the risk of MS [46]. Hypermethylation of all these methylation markers was significantly increased in MS patients compared to healthy controls. Lower levels of LINE-1 methylation were associated with lower EDSS scores in MS patients, while Alu showed higher level of methylation in the group of patients with low EDSS score [46]. No significant effect of methylation levels was observed on the number of relapses, the presence of spinal cord relapse, the age of MS onset and the presence of oligoclonal bands [46]. The knowledge of these repetitive elements across the entire genome is very limited, although it has already been proposed that LINE-1 hypermethylation could be the consequence of this DNMT upregulation [47].

## Concluding remarks—emerging issues

Epigenetics, and in particular DNA methylation, may be the bridge between genotypes, environmental exposures, and MS. However, studies on DNA methylation in MS are relatively few, with limited sample size, and perhaps, it is too early to draw firm conclusions so far. Yet, what is clear from the studies is that DNA methylation is influenced by environmental factors and affects gene expression that may predispose to MS. An additional contribution of studies of MS epigenetics is that they have revealed the significance of genetic loci that were not previously linked to MS [45].

Epigenetic findings in MS generally differed among different studies. A number of explanations may account for the discordant findings. Tissue specificity in DNA methylation, the epigenetic changes induced as a result of aging, the possible inability of detecting loci with low methylation changes, the limited sample size, the diversity in methodology, and the tested MS clinical phenotypes could explain, at least in part, the discordance of results among the studies [3, 31]. Purified cell populations should be preferred from mixtures of cells to receive tissue specific epigenetic profile. The use of new techniques could also help to identify and estimate epigenetic changes in vivo [3, 48]. Moreover, it should be examined how changes in DNA methylation influence the expression of the corresponding translational peptide. Routinely collected data in epidemiological studies could be considered as co-predictors of DNA methylation, as they have been reported to influence DNA methylation in healthy population [49]. In addition, prospective studies would reveal changes in DNA methylation years before the development of MS [50] and could provide us more information regarding a possible clinical application of DNA methylation as MS biomarker. The identification of such a biomarker in relation to disease development, clinical course or treatment response in patients with MS would provide physicians with a clinically useful and cost effective tool [34, 51, 52]. However, the role of methylation in MS needs further investigation as every genetic locus follows a different methylation pattern [28], and this is not necessarily linked to the development of the disease.

Epigenetic changes may be reversed by treatments intervening in the DNA methylation, histone deacetylation, and silencing of miRNAs [53, 54]. The search of epigenetic treatments, though, is still in its infancy. The current approach focuses on targeting key enzymes for the procedures of DNA methylation and histone deacetylation. Agents that inhibit DNMTs and histone deacetylase (such as 5-azacytidine and valproate, respectively) have been proved very effective in hematological cancers. However, clinical trials in MS patients have lingered, as the above-mentioned epigenetic processes have not yet been established as biomarkers for the development and severity of the disease. Another deterrent could be the lack of specificity of such agents, thus increasing the risk of side effects in MS patients. Although in vitro studies and trials in mouse models of MS seem to be very encouraging about the therapeutic potential of DNMT and histone deacetylase inhibitors, the contradictions mentioned should be taken into consideration [55]. Further research on epigenetics in MS is of great necessity to elucidate pathophysiological aspects and demonstrate more effective, riskless, and personalized therapeutic approaches.
